# Offending, custody and opioid substitution therapy treatment utilisation among opioid-dependent people in contact with the criminal justice system: comparison of Indigenous and non-Indigenous Australians

**DOI:** 10.1186/1471-2458-14-920

**Published:** 2014-09-06

**Authors:** Natasa Gisev, Amy Gibson, Sarah Larney, Jo Kimber, Megan Williams, Anton Clifford, Michael Doyle, Lucy Burns, Tony Butler, Don J Weatherburn, Louisa Degenhardt

**Affiliations:** National Drug and Alcohol Research Centre, UNSW Australia, Sydney, New South Wales Australia; Centre for Health Research, University of Western Sydney, Sydney, New South Wales Australia; Alpert Medical School, Brown University, Providence, Rhode Island USA; School of Public Health and Community Medicine, UNSW Australia, Sydney, New South Wales Australia; School of Population Health, University of Queensland, Brisbane, Queensland Australia; The Kirby Institute, UNSW Australia, Sydney, New South Wales Australia; New South Wales Bureau of Crime Statistics and Research (BOCSAR), Sydney, NSW Australia; School of Population and Global Health, The University of Melbourne, Melbourne, Victoria Australia

**Keywords:** Indigenous population, Aboriginal and Torres Strait Islander people, Opioid-related disorders, Opioid substitution treatment, Crime, Prisons, Data linkage

## Abstract

**Background:**

Although Indigenous Australians are over-represented among heroin users, there has been no study examining offending, time in custody, and opioid substitution therapy (OST) treatment utilisation among Indigenous opioid-dependent (including heroin) people at the population level, nor comparing these to non-Indigenous opioid-dependent people. The aims of this study were to compare the nature and types of charges, time in custody and OST treatment utilisation between opioid-dependent Indigenous and non-Indigenous Australians in contact with the criminal justice system.

**Methods:**

This was a population-based, retrospective data linkage study using records of OST entrants in New South Wales, Australia (1985–2010), court appearances (1993–2011) and custody episodes (2000–2012). Charge rates per 100 person-years were compared between Indigenous and non-Indigenous Australians by sex, age and calendar year. Statistical comparisons were made for variables describing the cumulative time and percentage of follow-up time spent in custody, as well as characteristics of OST initiation and overall OST treatment utilisation.

**Results:**

Of the 34,962 people in the cohort, 6,830 (19.5%) were Indigenous and 28,132 (80.5%) non-Indigenous. Among the 6,830 Indigenous people, 4,615 (67.6%) were male and 2,215 (32.4%) female. The median number of charges per person against Indigenous people (25, IQR 31) was significantly greater than non-Indigenous people (9, IQR 16) (p < 0.001). Overall, Indigenous people were charged with 33.2% of the total number of charges against the cohort and 44.0% of all violent offences. The median percentage of follow-up time that Indigenous males and females spent in custody was twice that of non-Indigenous males (21.7% vs. 10.1%, p < 0.001) and females (6.0% vs. 2.9%, p < 0.001). The percentage of Indigenous people who first commenced OST in prison (30.2%) was three times that of non-Indigenous people (11.2%) (p < 0.001). Indigenous males spent less time in OST compared to non-Indigenous males (median percentage of follow-up time in treatment: 40.5% vs. 43.1%, p < 0.001).

**Conclusions:**

Compared to non-Indigenous opioid-dependent people, Indigenous opioid-dependent people in contact with the criminal justice system are charged with a greater number of offences, spend longer in custody and commonly initiate OST in prison. Hence, contact with the criminal justice system provides an important opportunity to engage Indigenous people in OST.

**Electronic supplementary material:**

The online version of this article (doi:10.1186/1471-2458-14-920) contains supplementary material, which is available to authorized users.

## Background

Substance use is a major social and health challenge for Indigenous peoples worldwide, and contributes to 20% of the disease burden (in disability adjusted life years) experienced by Aboriginal and Torres Strait Islander (Indigenous) Australians [1]. Illicit drug use accounts for 3% of the total disease burden, and is responsible for 4% of the disparity in health between Indigenous and non-Indigenous Australians [1]. Indigenous people are over-represented among samples of injecting drug users in Australia - despite comprising about 3% of the population [2], between 11% and 12% of respondents in a national survey of needle and syringe program attendees identified as Indigenous [3]. Similarly, studies in Australia and Canada have shown that Indigenous injecting drug users are over-represented in experiencing the harms associated with injecting drug use, including higher incidences of HIV and hepatitis C [4–6].

Indigenous people worldwide are known to have contact with the criminal justice system at disproportionately high levels [7, 8]. About one-quarter of the prisoner population in Australia are Indigenous [9]. The most common charges for Australian Indigenous prisoners are acts intended to cause injury (34%), unlawful entry with intent (16%), and offences against justice procedures (11%) [9]. In addition to being more likely to be arrested, charged and imprisoned, the frequency of criminal justice contact is higher among Indigenous people [10], and occurs at an earlier age than non-Indigenous people [11]. About three-quarters (77%) of Indigenous people in Australian prisons have previously been imprisoned, compared with 51% of non-Indigenous people [9].

The factors contributing to over-representation of Indigenous people in the criminal justice system are complex and often inter-related [10]. Indigenous people experienced major disruption to every aspect of health, social, economic, cultural and spiritual systems following colonisation, and inter-generational trauma is ongoing. The Royal Commission into Aboriginal Deaths in Custody in the early 1990s proposed that the over-representation of Indigenous people in prison was due to the combined effect of bias in the criminal justice system and Indigenous economic and social disadvantage [12]. The Royal Commission recommended that Indigenous economic, social and cultural disadvantage, as well as substance abuse, be addressed [12]. Currently, there is little evidence to support the idea that discrimination and racial bias in policing and court decisions explain the over-representation of Indigenous people in prison [10, 13]. After adjusting for sentence-related factors such as current and past offending, Indigenous people are no more likely to be issued longer sentences than non-Indigenous people in New South Wales (NSW) courts [14].

Alcohol abuse and illicit drug use are strongly associated with the likelihood and number of arrests Indigenous people have experienced in the last five years [15]. Opioid substitution therapy (OST – methadone or buprenorphine), the preferred treatment for opioid dependence, has shown to be beneficial in reducing heroin use [16, 17], other drug use, crime, HIV infection, exposure to viral hepatitis, and mortality [18]. However, maintaining retention in treatment is necessary in order to maximise OST treatment outcomes [19]. Higher legal severity (corresponding with more complex and severe criminal justice histories) have been associated with shorter retention in methadone treatment [20]. However, there are limited data comparing outcomes related to OST use between Indigenous and non-Indigenous people. Ethnic minority groups have been found to experience significant delays in admission to methadone programs and lower retention once in treatment [21, 22]. Similar findings have been observed among Aboriginal people in studies of people who inject drugs in Canada [23]. In contrast, an evaluation of OST provision in an urban Australian Aboriginal Health Service found that rates of treatment retention and heroin use reduction were equivalent to those observed in mainstream treatment programs [24]. There is therefore a clear need for further research to resolve these differences in study findings, given the potential benefits of treatment in this population.

To date, there has been no study examining offending, time in custody, and OST treatment utilisation among Indigenous opioid-dependent people at the population level, nor comparing these to non-Indigenous opioid-dependent people. Using a population-level cohort of opioid-dependent people in contact with the criminal justice system in NSW, Australia, the aims of this study (relating to three main themes), were to:

1. Determine the distribution, type and outcome of criminal charges for Indigenous and non-Indigenous Australians (offending);
2. Compare the charge histories of Indigenous and non-Indigenous Australians by sex, age and calendar year (offending);
3. Compare the cumulative time and percentage of follow-up time spent in custody among Indigenous and non-Indigenous Australians (custody);
4. Compare initiation of OST treatment, overall OST treatment utilisation, and the temporal relationship between age of first offence and first commencing OST treatment, between Indigenous and non-Indigenous Australians (treatment utilisation).

## Methods

### Study cohort

The population-level cohort was defined on the basis that individuals had a recorded history of opioid dependence (as evidenced by the receipt of OST), at least one criminal charge during the study period, and valid information regarding their Indigenous identity (n = 34,962).

### Data sources

Three administrative datasets were used to define the cohort in this study and to compare patterns of offending, time in custody and opioid-substitution therapy (OST) treatment utilisation between Indigenous and non-Indigenous Australians.

### The Pharmaceutical Drugs of Addiction System (PHDAS) dataset

The PHDAS is a comprehensive record of all people in NSW to whom pharmaceutical drugs of addiction were dispensed by authorised clinicians through the NSW Opioid Treatment Program since 1985. The PHDAS records each client’s full name, date of birth, sex, OST program entry and exit dates, the OST medicine received (buprenorphine or methadone), the approved prescriber, the treatment setting (community or prison) and the reason for program exit [25]. A new treatment program is recorded in the PHDAS when an application to prescribe OST is approved by the NSW Ministry of Health, or if a client changes their prescriber, OST medicine, or point of administration. A person can therefore have multiple treatment programs during a period of continuous dosing. Consistent with earlier studies, a continuous OST treatment program was defined where there was less than seven days between program exit dates and subsequent program entry dates [26, 27].

### The Re-offending Database (ROD)

The ROD is a database maintained by the NSW Bureau of Crime Statistics and Research (BOCSAR) that contains records of all finalised court appearances (i.e. all court matters that are completed and have an outcome) in the Local, District and Supreme Courts of NSW since 1994 and custody episodes from the NSW Department of Corrective Services from 2000. Specifically, the ROD was used to extract data relating to all offences occurring between 1 December 1993 and 31 December 2011 and custody episodes which occurred between 1 January 2000 and March 2012. The internal matching process of the ROD dataset has been previously validated and identified to have a specificity of 99.9% and a sensitivity of 93.8% [28].

### The National Death Index (NDI)

The NDI is a fully identified dataset held by the Australian Institute of Health and Welfare (AIHW) which contains mortality records (date, State, and causes of death) collected from each of the State and Territory Births, Deaths and Marriage Registers across Australia. Date of death was used to terminate the follow-up period for individuals who died prior to the end of coverage in the datasets.

### Definitions

Receipt of OST was used as a marker of opioid dependence, which is a clinical criterion for receiving treatment with OST in NSW. We assumed ongoing opioid dependence from first entry to OST to the end of follow-up, as it is known that opioid dependence is a chronic relapsing disorder with low remission rates [29, 30].

The term Indigenous in this paper refers to individuals who identify as Australian Aboriginal and/or Torres Strait Islander people. An individual was considered Indigenous if they were *ever-identified* as an Aboriginal and/or Torres Strait Islander person in either the ROD or PHDAS datasets (determined by self-report). Therefore, individuals with conflicting records (who were identified as Indigenous in one dataset and non-Indigenous in the other) were also considered as Indigenous. Records for people with an unknown/missing Indigenous identity were excluded from the cohort (n = 13,107).

Offending was evaluated using data relating to charges (recorded crime) and were coded according to the Australian and New Zealand Standard Offence Classification (ANZSOC) system which has 16 major categories of offences [31]. This study used definitions consistent with the BOCSAR’s standard crime and statistic reporting [32]. *Violent offences* included murder, assault, robbery, sexual assault and indecent assault/act of indecency/other sexual offences. *Property offences* included break and enter, motor vehicle theft, theft and fraud.

### Statistical analyses

Through the linkage of the datasets described, this study provided a unique opportunity to conduct longitudinal population-level analyses. All analyses were conducted using SAS Enterprise Guide 5.1 (SAS Institute Inc., Cary, NC, USA). Descriptive statistics were used to summarise charges, time in custody and OST treatment utilisation. Comparisons between Indigenous and non-Indigenous Australians in the cohort were made using chi-square tests of association for categorical variables and Wilcoxon-Mann-Whitney tests for continuous variables. Due to the different date ranges available for the each of the datasets, we defined distinct follow-up periods to determine the total observation period for each analysis, taking into account the date of death (if recorded).

### Offending

The follow-up time for the analyses of charges commenced on 1 December 1993, or whenever the individual turned 10 years of age (whichever was later); 10 years is the age of criminal responsibility in NSW. Follow-up ceased on 31 December 2011, or when death occurred (whichever was earlier). In addition to comparisons of offence frequencies, rates of charges per 100 person years (PY) were also calculated for Indigenous and non-Indigenous males and females, by age group, and calendar year to determine if rates varied across age groups and over time.

### Time in custody

The follow-up time for the analyses of custody episodes commenced on 1 January 2000, or whenever the participant turned 10 years of age (whichever was later). The follow-up time ceased on 31 March 2012, or when death occurred (whichever was earlier). Custody episodes where the individual was received and released on the same day were excluded. In calculating the duration of custody episodes, both complete and incomplete custody episodes were included. Complete custody episodes were those completely contained within the follow-up period (i.e. reception date of or after 1 January 2000 and release date of or before 31 March 2012). Incomplete custody episodes were those which started prior to January 1 2000 and/or had not ended by 31 March 2012. In these cases, only days within the follow-up period were counted. The percentage of total follow-up time each individual spent in custody was compared using the cumulative time spent in custody (including incomplete episodes) and the total length of follow-up (start of custody to 31 March 2012 or death).

### OST treatment utilisation

The follow-up time for analyses relating to OST treatment utilisation commenced on the day of OST initiation (with records from 1 January 1985) and ended at death or the date of data extraction (18 May 2012), whichever was earlier. Individuals who had commenced treatment and had no treatment end date listed, were deemed to still be receiving treatment at the date of extraction. Treatment retention was evaluated at three, six, nine and 12 months after first commencing OST and compared between Indigenous and non-Indigenous offenders. The percentage of total follow-up time each individual spent in treatment was compared using the cumulative time spent in treatment and the total length of follow-up (start of treatment to 18 May 2012 or death).

### Temporal relationship between age of first offence and first commencing OST treatment

In order to examine the temporal relationship between age of first charge and first commencing OST treatment, we identified a sub-set of individuals from the total study population who were below the age of criminal responsibility in NSW (10 years) at the beginning of the charges dataset (1 December 1993) (n = 2,815). We therefore had their complete criminal charge histories and were able to analyse these in relation to their first OST commencement.

### Ethical approval

Ethical approval to conduct this study was obtained from the ethics committees of the NSW Aboriginal Health and Medical Research Council (AH&MRC), University of New South Wales, NSW Health’s Population & Health Services Research Ethics Committee, the AIHW, the Alfred Hospital (Victoria), Corrective Services NSW, Justice Health and Forensic Mental Health Network (NSW Health), and the Department of Justice (Victoria).

## Results

Of the 34,962 individuals in the cohort, 6,830 (19.5%) were Indigenous; 4,615 (67.6%) were male and 2,215 (32.4%) were female (Table 1). Among the 28,132 individuals in the cohort who were non-Indigenous, 20,179 (71.7%) were male and 7,953 (28.3%) were female (p < 0.001).

**Table 1:** **Charge histories of opioid-dependent people with at least one criminal charge according to Indigenous status, December 1993-December 2011**

Among people charged at least once	Indigenous (N = 6,830)	Non-Indigenous (N = 28,132)	Total (N = 34,962)
Males	4,615 (67.6%)	20,179 (71.7%)	24,794 (70.9%)
Females	2,215 (32.4%)	7,953 (28.3%)*	10,168 (29.1%)
Median no. charges (IQR) (Min-Max)	25 (31) (1–175)	9 (16) (1–314)#	11 (21) (1–314)
Median no. proven charges (IQR) (Min-Max)			
Among all charged	21 (27) (0–166)	8 (15) (0–301)#	9 (18) (0–301)
Among those who had any proven charge	21 (27) (1–166)	8 (15) (1–301) #	10 (18) (1–301)
N (%) charges by major crime types			
Homicide and related offences	144 (0.1%)	262 (0.1%)	406 (0.1%)
Acts intended to cause injury	27,647 (13.1%)	35,490 (8.4%)	63,137 (10.0%)
Sexual assault and related offences	590 (0.3%)	739 (0.2%)	1,329 (0.2%)
Dangerous or negligent acts endangering persons	3,834 (1.8%)	10,498 (2.5%)	14,344 (2.3%)
Abduction, other offences against the person	1,204 (0.6%)	1,597 (0.4%)	2,801 (0.4%)
Robbery, extortion and related offences	3,010 (1.4%)	3,554 (0.8%)	6,564 (1.0%)
Unlawful entry/burglary, break and enter	13,169 (6.3%)	20,347(4.8%)	33,516 (5.3%)
Theft and related offences	50,364 (23.9%)	105,453 (24.9%)	155,817(24.6%)
Fraud, deception and related offences	7,275 (3.5%)	23,076 (5.4%)	30,351 (4.8%)
Illicit drug offences	15,132 (7.2%)	47,903 (11.3%)	63,035 (9.9%)
Prohibited and regulated weapons offences	1,780 (0.9%)	5,111 (1.2%)	6,891 (1.1%)
Property damage and environmental pollution	9,676 (4.6%)	12,964 (3.1%)	22,640 (3.6%)
Public order offences	21,629 (10.3%)	34,999 (8.3%)	56,628 (8.9%)
Traffic and vehicle regulatory offences	27,231 (12.9%)	75,682 (17.8%)	102,913 (16.2%)
Offences against justice procedures	26,076 (12.4%)	40,940 (9.7%)	67,016 (10.6%)
Miscellaneous offences	1,832 (0.9%)	5,588 (1.3%)	7,420 (1.2%)
Charges for any property offence (N, % all charges)	52,594 (25.0%)	104,930 (25.0%)	157,524 (25.0%)
Charges for any violent offence (N, % all charges)	31,349 (14.9%)	39,962 (9.4%)	71,311 (11.2%)
Total number of charges	210,593	424,203	634,796
Total number of proven charges	177,887	365,531	543,418
Percentage of charges proven	84.5%	86.2%	85.6%

*Chi-square test, p < 0.001; # Wilcoxon-Mann-Whitney test, p < 0.001.

### Offending

**Figure 1:**
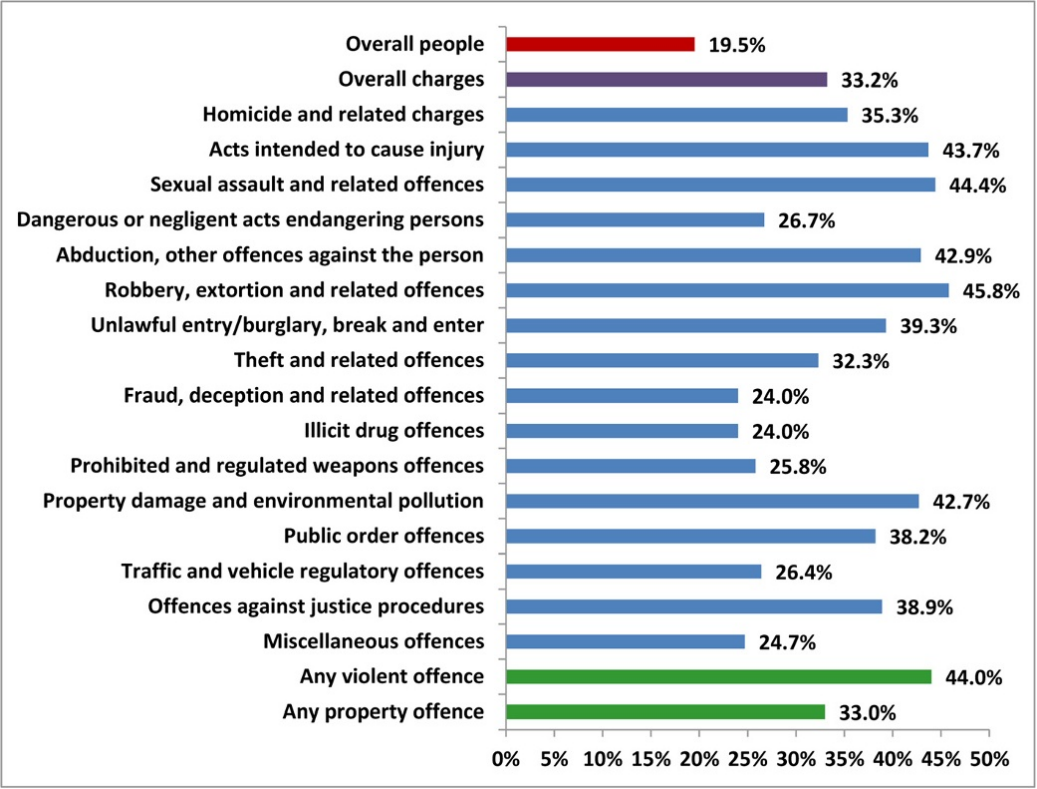
**The proportion of charges laid against Indigenous offenders, December 1993- December 2011.**

A total of 210,593 charges were laid against Indigenous offenders, and 424,203 charges were laid against non-Indigenous offenders (Table 1). The median number of charges for Indigenous offenders (25, IQR 31) was almost three times that of non-Indigenous offenders (9, IQR 16) (p < 0.001), indicating that repeat offending was common. The percentage of charges which were proven (i.e. those with a guilty verdict) was similar between Indigenous and non-Indigenous males (83.5% vs. 85.6%) and females (87.3 vs. 88.4%) (Additional file 1). Charges for theft and related offences were the most prevalent, representing 23.9% of all charges by Indigenous offenders and 24.9% of all charges by non-Indigenous offenders. Property offences represented 25.0% of all charges for both Indigenous and non-Indigenous offenders. However, the percentage of charges for a violent offence was greater among Indigenous offenders (14.9%) than non-Indigenous offenders (9.4%). Between 68.7% (males) and 73.5% (females) of charges for violent offences by non-Indigenous people were proven. Similarly, between 69.6% (males) and 76.3% (females) of charges for violent offences by Indigenous people were proven.Although Indigenous offenders comprised 19.5% of the cohort, they contributed to 33.2% of the total charges laid against the cohort; ranging between 24.0% (fraud and illicit drug offences) and 45.8% (robbery and related offences) of specific charge types (Figure 1). Considering all violent offences, Indigenous offenders contributed to almost half (44.0%) of all violent offence charges laid against the cohort.

**Figure 2:**
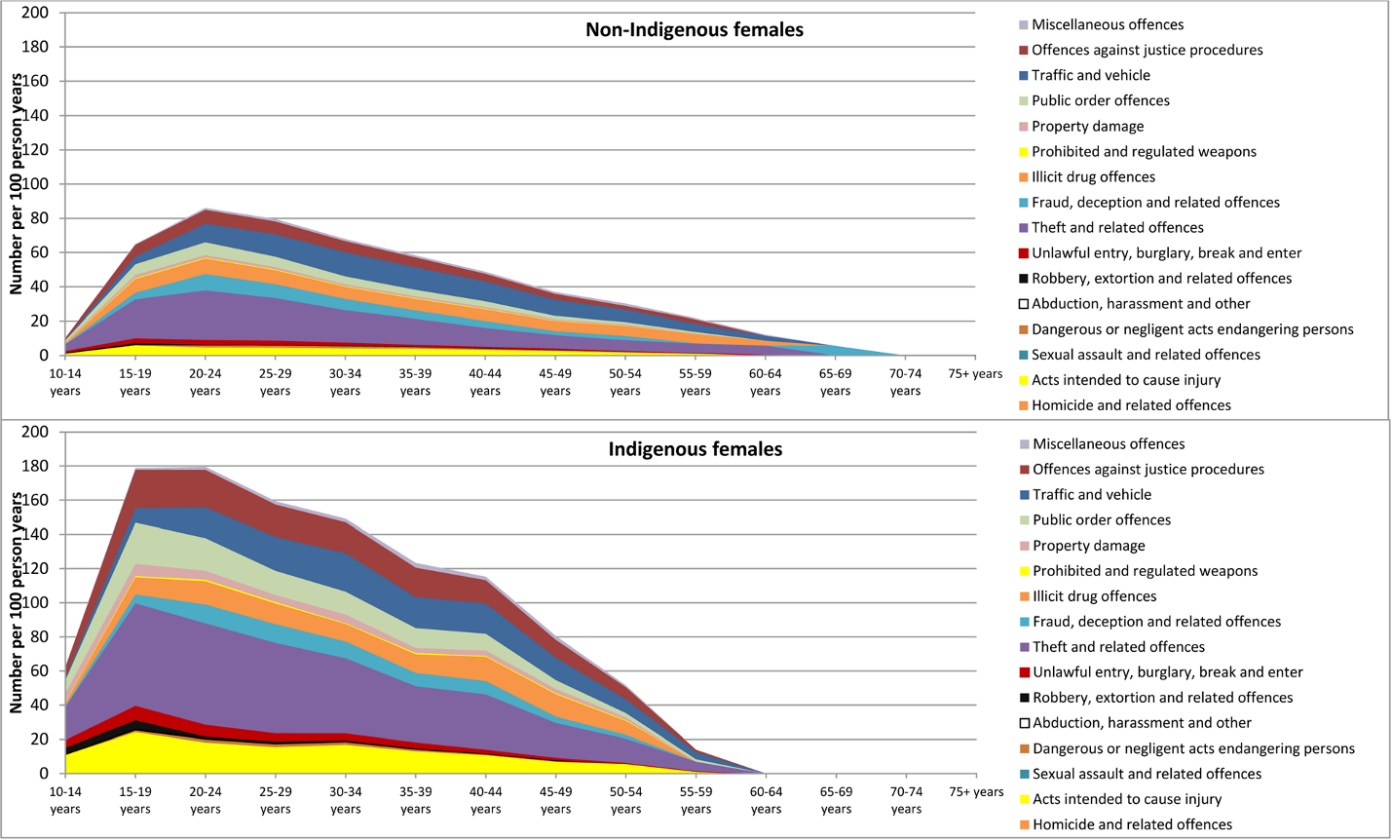
**Number of charges against opioid-dependent people per 100 person years for non-Indigenous and Indigenous females, according to type of offence and age group.**

**Figure 3:**
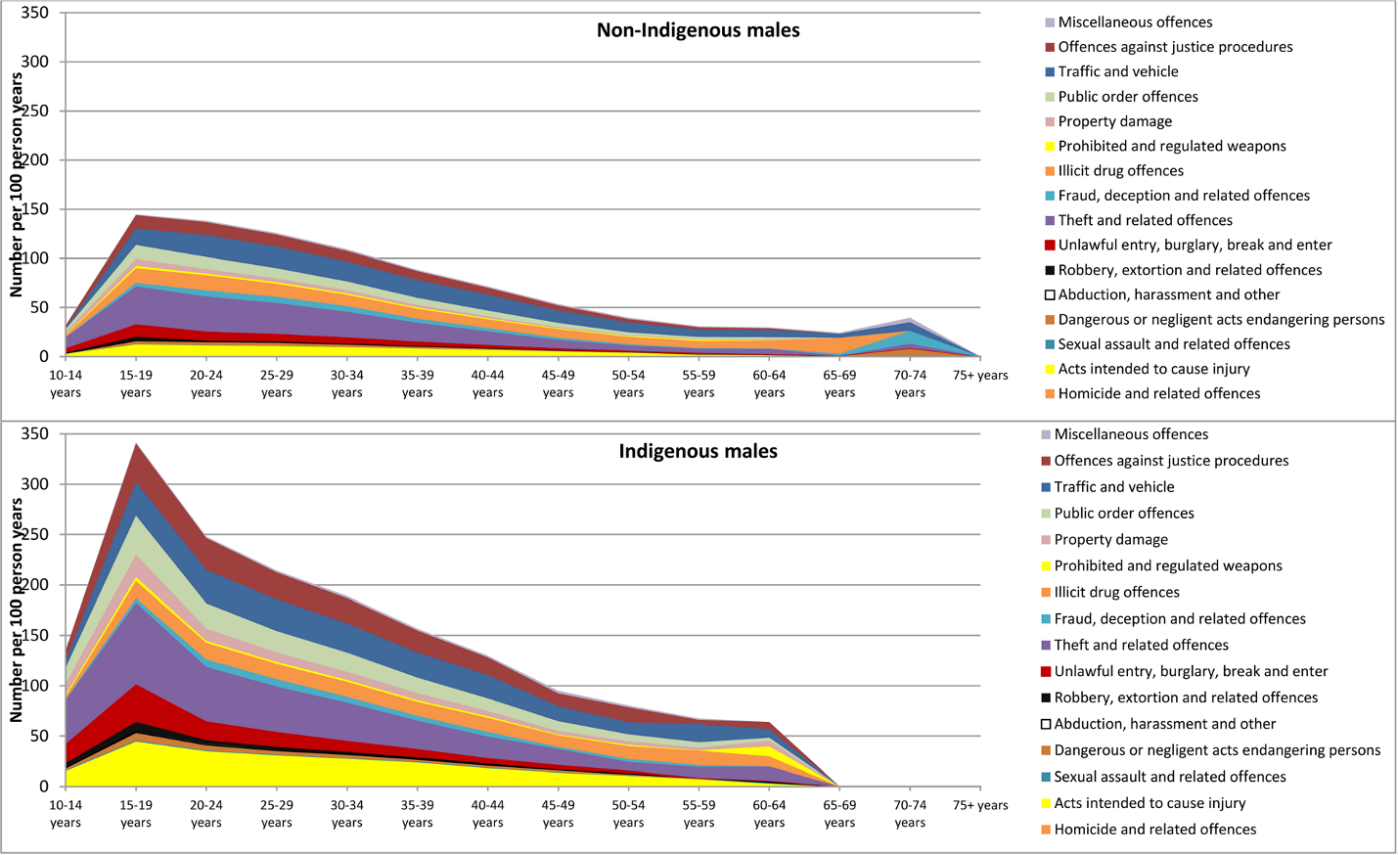
**Number of charges against opioid-dependent people per 100 person years for non-Indigenous and Indigenous males, according to type of offence and age group.**

The distribution and outcomes of charges laid against Indigenous and non-Indigenous males and females are shown in Table 2. Among male offenders, the largest difference in the percentage of charges laid against Indigenous and non-Indigenous people were for offences relating to property damage and environmental pollution such as noise, air or water pollution (61.1% vs. 29.0%, p < 0.001), followed by offences against justice procedures (80.3% vs. 49.9%, p < 0.001). Among female offenders, the largest difference in the percentage of charges laid against Indigenous and non-Indigenous people were for offences relating to acts intended to cause injury (62.4% vs. 29.3%, p < 0.001), followed by offences against justice procedures (69.8% vs. 37.8%, p < 0.001).Figures 2 and 3 show that across age categories, charge rates (number of charges/100 person-years) were approximately two times greater among female and male Indigenous offenders than non-Indigenous female and male offenders, and were higher among younger age groups. The charge rate among non-Indigenous females peaked at 20–24 years of age (86.1 charges/100 person-years), whereas for Indigenous females, the charge rate was highest across two age categories - 15–19 years (178.9 charges/100 person-years) and 20–24 years (179.6 charges/100 person-years). Conversely, charge rates for both Indigenous and non-Indigenous males peaked at 15–19 years of age (341.8 charges/100 person-years and 144.7 charges/100 person-years, respectively). There was an overall decline in charge rate with increasing age for both males and females.

**Table 2:** **Outcomes of charges against opioid-dependent people with at least one criminal charge by Indigenous status and sex, December 1993-December 2011**

	Males (N = 24,794)						Females (N = 10,168)					
	Charged			Proven			Charged			Proven		
	Indigenous	Non-Indigenous	P*	Indigenous	Non-Indigenous	P*	Indigenous	Non-Indigenous	P*	Indigenous	Non-Indigenous	P*
	(N = 4,615)	(N = 20,179)		(N = 4,615)	(N = 20,179)		(N = 2,215)	(N = 7,953)		(N = 2,215)	(N = 7,953)	
Offence type	N (%)	N (%)		N (%)	N (%)		N (%)	N (%)		N (%)	N (%)	
Homicide and related offences	93	172	<0.001	56	105	<0.001	21	24	<0.001	15	17	0.001
	(2.0%)	(0.9%)		(1.2%)	(0.5%)		(0.9%)	(0.3%)		(0.7%)	(0.2%)	
Acts intended to cause injury	3,655	9,585	<0.001	3,386	8,273	<0.001	1,383	2,329	<0.001	1,279	2,001	<0.001
	(79.2%)	(47.5%)		(73.4%)	(41.0%)		(62.4%)	(29.3%)		(57.7%)	(25.2%)	
Sexual assault and related offences	280	369	<0.001	182	219	<0.001	18	18	<0.001	11	7	N/A
	(6.1%)	(1.8%)		(3.9%)	(1.1%)		(0.8%)	(0.2%)		(0.5%)	(0.1%)	
Dangerous or negligent acts endangering persons	1,597	5,222	<0.001	1,497	4,874	<0.001	357	1,191	0.186	338	1,117	0.149
	(34.6%)	(25.9%)		(32.4%)	(24.2%)		(16.1%)	(15.0%)		(15.3%)	(14.0%)	
Abduction, other offences against the person	694	1,026	<0.001	564	788	<0.001	124	105	<0.001	90	71	<0.001
	(15.0%)	(5.1%)		(12.2%)	(3.9%)		(5.6%)	(1.3%)		(4.1%)	(0.9%)	
Robbery, extortion and related offences	1,074	1,631	<0.001	897	1,375	<0.001	286	246	<0.001	228	202	<0.001
	(23.3%)	(8.1%)		(19.4%)	(6.8%)		(12.9%)	(3.1%)		(10.3%)	(2.5%)	
Unlawful entry/burglary, break and enter	2,597	5,694	<0.001	2,383	5,029	<0.001	685	988	<0.001	580	802	<0.001
	(56.3%)	(28.2%)		(51.6%)	(24.9%)		(30.9%)	(12.4%)		(26.2%)	(10.1%)	
Theft and related offences	3,928	13,209	<0.001	3,793	12,615	<0.001	1,862	5,138	<0.001	1,826	4,914	<0.001
	(85.1%)	(65.5%)		(82.2%)	(62.5%)		(84.1%)	(64.6%)		(82.4%)	(61.8%)	
Fraud, deception and related offences	1,529	5,564	<0.001	1,435	5,236	<0.001	837	2,174	<0.001	804	2,063	<0.001
	(33.1%)	(27.6%)		(31.1%)	(25.9%)		(37.8%)	(27.3%)		(36.3%)	(26.0%)	
Illicit drug offences	3,231	12,382	<0.001	3,159	12,066	<0.001	1,319	3,827	<0.001	1,282	3,688	<0.001
	(70.0%)	(61.4%)		(68.5%)	(59.8%)		(59.5%)	(48.1%)		(57.9%)	(46.4%)	
Prohibited, regulated weapons offences	894	2,683	<0.001	779	2,328	<0.001	178	361	<0.001	153	292	<0.001
	(19.4%)	(13.3%)		(16.9%)	(11.5%)		(8.0%)	(4.5%)		(6.9%)	(3.7%)	
Property damage, environmental pollution	2,820	5,853	<0.001	2,626	5,215	<0.001	811	1,168	<0.001	743	1,018	<0.001
	(61.1%)	(29.0%)		(56.9%)	(25.8%)		(36.6%)	(14.7%)		(33.5%)	(12.8%)	
Public order offences	3,601	9,764	<0.001	3,462	9,085	<0.001	1,424	2,696	<0.001	1,370	2,515	<0.001
	(78.0%)	(48.3%)		(75.0%)	(45.0%)		(64.3%)	(33.9%)		(61.9%)	(31.6%)	
Traffic and vehicle regulatory offences	3,316	12,505	<0.001	3,284	12,314	<0.001	1,233	3,844	<0.001	1,214	3,778	<0.001
	(71.9%)	(62.0%)		(71.2%)	(61.0%)		(55.7%)	(48.3%)		(54.8%)	(47.5%)	
Offences against justice procedures	3,706	10,073	<0.001	3,559	9,398	<0.001	1,547	3,004	<0.001	1,471	2,738	<0.001
	(80.3%)	(49.9%)		(77.1%)	(46.6%)		(69.8%)	(37.8%)		(66.4%)	(34.4%)	
Miscellaneous offences	758	2,714	<0.001	675	2,367	<0.001	428	936	<0.001	380	819	<0.001
	(16.4%)	(13.4%)		(14.6%)	(11.7%)		(19.3%)	(11.8%)		(17.2%)	(10.3%)	
Any property offence	3,894	12,754	<0.001	3,789	12,236	<0.001	1,821	4,990	<0.001	1,781	4,803	<0.001
	(84.4%)	(63.2%)		(82.1%)	(60.6%)		(82.2%)	(62.7%)		(80.4%)	(60.4%)	
Any violent offence	3,773	10,137	<0.001	3,539	8,856	<0.001	1,434	2,440	<0.001	1,330	2,113	<0.001
	(81.8%)	(50.2%)		(76.7%)	(43.9%)		(64.7%)	(30.7%)		(60.0%)	(26.6%)	
Total	33,773	98,446	--	31,737	91,287	--	12,513	28,049	--	11,784	26,042	--

*p-values calculated using Chi-square tests for a 2 × 2 table for each crime type – number of Indigenous and non-Indigenous offenders charged/proven vs not charged/proven.

The charge rates (number of charges/100 person-years) between non-Indigenous and Indigenous males and females across the 1993–2011 calendar years are shown in Additional files 2 and 3. For each group, charge rates were relatively consistent across all years, with the exception of a clear peak in overall charge rates in 2001, corresponding with the time that a heroin shortage was observed across Australia [33, 34].

### Time in custody

A total of 17,967 individuals spent at least one full day in custody between January 2000 and March 2012, with Indigenous people representing 5,303 (29.5%) of all those who were incarcerated among the cohort (Table 3). Overall, Indigenous people spent twice the median percentage of follow-up time in custody compared with non-Indigenous people (21.7% vs. 10.1%, p < 0.001 for males, and 6.0% vs. 2.9%, p < 0.001 for females). This was both the result of Indigenous people having more custody episodes (median of 5 episodes for males and 4 for females), and episodes were of a longer duration (median 75 days for males and 30 days for females). In comparison, the median number of custody episodes for non-Indigenous people was 3 episodes for males and 2 episodes for females; the median duration of custody episodes was 68 days for males and 22 days for females.

**Table 3:** **Profile of custody episodes** ^**1**^ **for 17,967 opioid-dependent people according to Indigenous status, January 2000 – March 2012**

	Males (N = 14,012)					Females (N = 3,746)				
	Indigenous		Non-Indigenous			Indigenous		Non-Indigenous		
	(N = 3,854)		(N = 10,158)			(N = 1,449)		(N = 2,297)		
	Median	IQR Min-Max	Median	IQR Min-Max	***P***	Median	IQR Min-Max	Median	IQR Min-Max	***P***
Number of custody episodes	5	6	3	4	<0.001	4	5	2	3	<0.001
		1-47		1-39			1-35		1-26	
Duration of custody episodes (days)	75	206	68	205	0.004	30	119	22	108	<0.001
		1–6,819		1–8,348			1–5,201		1–5,341	
Percentage of follow-up time each individual spent in custody	21.7	38.2	10.1	24.0	<0.001	6.0	16.1	2.9	8.8	<0.001
		0.02-100		0.02-100			0.02-100		0.02-100	

^1^Includes incomplete episodes, i.e. days in the follow-up period spent in custody for episodes that had started prior to 2000, and/or which had not ended by March 2012.

### OST treatment utilisation

Table 4 compares OST treatment utilisation during the first treatment episode and in total over the follow-up period, between Indigenous and non-Indigenous males and females. More Indigenous offenders first commenced OST while in custody than non-Indigenous offenders (30.2% vs. 11.2%, p < 0.001), which was consistent among both males (37.7% vs. 13.8%, p < 0.001) and females (14.8% vs. 4.6%, p < 0.001).

**Table 4:** **OST treatment utilisation among opioid-dependent offenders, by Indigenous status and sex, January 1985 – December 2010**

	Males (N = 24,794)					Females (N = 10,168)					Total (N = 34,962)				
	Indigenous		Non-Indigenous			Indigenous		Non-Indigenous			Indigenous		Non-Indigenous		
	N = 4,615		N = 20,179			N = 2,215		N = 7,953			N = 6,830		N = 28,132		
	n	%	n	%		n	%	n	%		n	%	n	%	
	(Median)	(Min-Max)	(Median)	(Min-Max)	***P***	(Median)	(Min-Max)	(Median)	(Min-Max)	***P***	(Median)	(Min-Max)	(Median)	(Min-Max)	***P***
**First treatment episode**															
*Age at treatment entry (years):*	26.4	9.1 (IQR)	27.7	9.7 (IQR)	<0.001	24.3	18.6 (IQR)	25.9	9.5 (IQR)	<0.001	25.7	9.0 (IQR)	27.2	9.7 (IQR)	<0.001
Median IQR (min-max)		14.3-60.6		14.9-73.3			14.8-49.2		14.2-63.9			14.3-60.6		14.2-73.3	
*OST first received:*					<0.001					0.168					<0.001
Methadone	4,004	86.8	16,700	82.8		1,909	86.2	6,761	85.0		5,913	86.6	23,461	83.4	
Buprenorphine	611	13.2	3,479	17.2		306	13.8	1,192	15.0		917	13.4	4,671	16.6	
*Setting:*					<0.001					<0.001					<0.001
Community	2,877	62.3	17,403	86.2		1,888	85.2	7,585	95.4		4,765	69.8	24,988	88.8	
Prison	1,738	37.7	2,776	13.8		327	14.8	368	4.6		2,065	30.2	3,144	11.2	
*Year of treatment entry:*					<0.001					<0.001					<0.001
1985–1990	353	7.7	3,001	14.9		266	12.0	1,342	16.9		619	9.1	4,343	15.4	
1991–1995	587	12.7	3,648	18.1		337	15.2	1,453	18.3		924	13.5	5,101	18.1	
1996–2000	1,204	26.1	5,991	29.7		645	29.1	2,418	30.4		1,849	27.1	8,409	29.9	
2001–2005	1,212	26.3	4,438	22.0		568	25.6	1,690	21.3		1,780	26.1	6,128	21.8	
2006-2010	1,259	27.3	3,101	15.4		399	18.0	1,050	13.2		1,658	24.3	4,151	14.8	
*Duration of episode (days):*	169	560 (IQR)	206	776 (IQR)	<0.001	177	657 (IQR)	288	1,001 (IQR)	<0.001	172	595 (IQR)	227	843 (IQR)	<0.001
Median IQR (min-max)		1–9,518		1–9,980			1–9,752		2–9,915			1-9752		1-9980	
*Number of people in treatment at:*															
3 months	2,466	53.4	11,496	57.0	<0.001	1,216	54.9	5,001	62.9	<0.001	3,682	53.9	16,497	58.6	<0.001
6 months	1,793	38.9	8,807	43.6	<0.001	905	40.9	3,492	49.6	<0.001	2,698	39.5	12,749	45.3	<0.001
9 months	1,378	29.9	7,190	35.6	<0.001	711	32.1	3,253	40.9	<0.001	2,089	30.6	10,443	37.1	<0.001
12 months	1,106	24.0	6,050	30.0	<0.001	584	26.4	2,764	34.8	<0.001	1,690	24.7	8,814	31.3	<0.001
**Overall treatment utilisation**															
*Form of OST ever received:*					<0.001					<0.001					<0.001
Methadone only	2,881	62.4	12,136	60.1		1,348	60.9	5,032	63.3		4,229	61.9	17,168	61.0	
Buprenorphine only	272	5.9	2,034	10.1		124	5.6	608	7.6		396	5.8	2,642	9.4	
Methadone and buprenorphine	1,462	31.7	6,009	29.8		743	33.5	2,313	29.1		2,205	32.3	8,322	29.6	
*Number of OST switches within a treatment episode:*					<0.001					0.001					<0.001
0	3,153	68.3	14,170	70.2		1,472	66.5	5,640	70.9		4,625	67.7	19,810	70.4	
1–5	1,391	30.1	5,830	28.9		717	32.4	2,236	28.1		2,108	30.9	8,066	28.7	
>5	71	1.5	179	0.9		26	1.2	77	1.0		97	1.4	256	0.9	
*Ever received OST in prison:*					<0.001					<0.001					<0.001
No	1,492	32.3	12,470	61.8		1,136	51.3	6,314	74.4		2,628	38.5	18,784	66.8	
Yes	3,123	67.7	7,709	38.2		1,079	48.7	1,639	20.6		4,202	61.5	9,348	33.2	
*Total number of treatment episodes:*	2	3 (IQR)	2	3 (IQR)	0.348	3	4 (IQR)	2	3 (IQR)	<0.001	2	3 (IQR)	2	3 (IQR)	<0.001
Median IQR (min-max)		1-23		1-30			1-25		1-26			1-25		1-30	
*Percentage of follow-up time each individual spent in treatment:* ^*#*^	40.5	58.3 (IQR)	43.1	66.6 (IQR)	0.002	57.9	59.5 (IQR)	55.8	66.5 (IQR)	0.885	45.6	60.2 (IQR)	46.5	67.3 (IQR)	0.052
Median IQR (min-max)		0.02-100		0.01-100			0.04-100		0.02-100			0.02-100		0.01-100	

^#^For each individual, the cumulative time spent in treatment as a proportion of the total length of follow-up (start of treatment to 18 May 2012 or death) multiplied by 100.

Examining treatment retention during individuals’ first OST treatment episode, the percentage of people in treatment at three, six, nine and 12 months was lower among Indigenous offenders (decreasing from 53.9% at three months to 24.7% at 12 months) than non-Indigenous offenders (decreasing from 58.6% at three months to 31.3% at 12 months). Furthermore, the lower treatment retention observed among Indigenous offenders was consistent for both males (decreasing from 53.4% at three months to 24.0% at 12 months) and females (decreasing from 54.9% at three months to 26.4% at 12 months).

Although there was no difference in the median number of OST treatment episodes between Indigenous (2, IQR 3) and non-Indigenous (2, IQR 3) males (p = 0.348), the median number of treatment episodes was greater for Indigenous females (3, IQR 4) than non-Indigenous females (2, IQR 3) (p < 0.001). Indigenous males spent less time in treatment over the follow-up period compared to non-Indigenous males (40.5% vs. 43.1%, p = 0.002). There was no difference in time in treatment among Indigenous females compared to non-Indigenous females (57.9% vs. 55.8%, p = 0.885).

The temporal relationship between age of first offence and first commencing OST treatment among the sub-cohort of 2,815 people with full offending data available is examined in Additional file 4. Among males, the median age of first OST entry among Indigenous offenders was 6.6 years after their first charge, compared to 3.9 years among non-Indigenous offenders, a difference of 2.7 years. Similarly, among females, the median age of first OST entry among Indigenous offenders was 4.2 years after their first offence, compared to 1.5 years among non-Indigenous offenders.

## Discussion

The results from this study provide the first population-level comparison of offending, time in custody, and OST treatment utilisation among opioid-dependent Indigenous and non-Indigenous Australians in contact with the criminal justice system.

### Offending

Despite comprising less than one fifth of the cohort, Indigenous Australians accounted for one third of all charges, indicating that disproportionately more charges were laid against Indigenous people in the cohort, and that repeat offending was common; a finding consistent with previous studies [10, 35, 36]. Theft, traffic offences and illicit drug offences were the three most common offence types among non-Indigenous males and females. Conversely, among Indigenous males and females, theft, acts intended to cause injury, and offences against justice procedures (e.g. failing to appear before court) were most common. Based on 2012 estimates, acts intended to cause injury, unlawful entry with intent, and offences against justice procedures accounted for almost 60% of offences recorded among Indigenous Australians [37]. Therefore, compared to the broader population of Indigenous offenders in Australia, theft was more common among our cohort. Acquisitive crime is known to be higher among people who use drugs as it has the potential to generate income to support their drug use [38], a key factor likely to be responsible for the higher rate of theft and related offences observed in our cohort. In addition, a history of criminal arrest may be a barrier to gaining employment, resulting in further criminal activity in order to generate income. It has previously been estimated that about 15% of the difference in employment-population rates between Indigenous and non-Indigenous Australians is due to the difference in arrest rates [39]. Indigenous Australians historically experienced higher unemployment rates, lower income and inter-generational poverty than other Australians, and fare worse across other related social determinants of health [40]. Also relevant to our cohort, was the peak in the rate of charges that occurred in 2001; a direct consequence of the heroin shortage that occurred in Australia (most notably in NSW) during that time [34]. The shortage led to an increase in the price of heroin, which subsequently resulted in an increase in the rate of acquisitive crime [33].

Indigenous people contributed to almost half (44%) of all violent-related offences. The higher rate of violent offending among Indigenous people is well documented [41]. In addition, compared to non-Indigenous violent offenders, Indigenous violent offenders are more likely to be re-incarcerated for a violent offence, and within shorter periods of time [41]. Multiple inter-related factors embedded in the historical experiences of Indigenous people are likely to contribute to their high rates of violent offending, including for example, loss of land and culture, trans-generational trauma, grief and loss, and social exclusion [42]. Although alcohol use is often a major factor implicated in violent crimes [41, 43], the relationship between illicit drug use and violent crime is less clear. In national surveys of police detainees and prisoners, Indigenous offenders were less likely than non-Indigenous offenders to self-report heroin use in the 30 days prior to being detained by police, and also in the six months prior to imprisonment [11]. However, in a survey of drug use and crime behaviour among male offenders incarcerated in prison, drug markets were identified as being associated with high levels of violence [44], with the highest being among those whose preferred drug of choice was heroin - 29% of respondents reported using force or threats of violence and 17% reported using weapons to obtain heroin. Hence, despite the various factors influencing Indigenous people to commit violent crimes, the role of illicit drug use cannot be excluded.

Previous studies have identified that Indigenous male offenders tend to have earlier and more serious contact with the criminal justice system [11]. In our cohort, offending commenced about three years earlier among Indigenous people. Indigenous females in particular, had charge rates that peaked earlier and for longer (15–19 years to 20–24 years) compared to non-Indigenous females (20–24 years). Conversely, charge rates peaked between 15–19 years for both Indigenous and non-Indigenous males. To date, offending patterns among Indigenous men have been more widely studied than that of Indigenous women [45, 46]. Current evidence suggests that discrimination among Indigenous women is compounded by being both female and Indigenous, and that few criminal justice system services and interventions are targeted to their needs [46]. Most services are either designed for Indigenous men, or for women in general, and are not culturally specific [41, 46]. Consequently, our results highlight that there is a great need to further understand and address Indigenous females’ over-representation in the criminal justice system.

There was also a clear reduction in the rate of offending with increasing age, particularly among Indigenous people. The low offending rates observed beyond 65 years for females and 69 years for males is likely to be related to poorer health, and multiple co-morbidities experienced by older Indigenous people, and life expectancy being only 72.9 for females and 67.2 for males - on average 13 years less than non-Indigenous Australians [47].

### Time in custody

Indigenous people represented 30% of all of people who were incarcerated among our cohort, whereas the point-prevalent estimate of the percentage of Indigenous people in prison in NSW in 2013 is 23% [9]. Given that Indigenous people comprise 2.9% of the general population in NSW [2], our findings suggest that much of the over-representation of Indigenous people in custody may be due to greater contact among people who are opioid dependent. Previously we have shown that there are marked differences in the cumulative time spent in custody among Indigenous and non-Indigenous opioid-dependent people [48]. In this study, Indigenous people spent twice the median percentage of follow-up time in custody, a consequence of these individuals having more custody episodes, as well as episodes being of a longer duration. This was consistent for both males and females. Over the twelve year period between 2000 and 2011, there was a 62% increase in the age-standardised imprisonment rate among Indigenous people in Australia [25]. In comparison, a marginal increase of 5% was observed among non-Indigenous people, highlighting the extent of disadvantage experienced by Indigenous people in contact with the criminal justice system [25]. Periods of imprisonment carry many health risks for people who inject drugs due to needle sharing and transmission of blood borne viruses [49, 50], potentiating risks which are already greater among Indigenous people [5]. Therefore, encouraging the use of OST to reduce the health risks associated with injecting drug use is especially important among Indigenous people who are imprisoned.

### OST treatment utilisation

Recent data show that Indigenous people are over-represented in OST in NSW [25]. We found that Indigenous people more frequently commenced OST in custody, which suggests under-treatment in the community. Although not directly evaluated in our study, there is evidence that OST treatment is associated with reduced offending [51–53]. Given that Indigenous people in our cohort also spent significantly more time in custody, these findings highlight the importance of making OST accessible through prisons. However, due to different OST policies and programs across countries and jurisdictions, access to OST in prisons vary widely [54]. For example, in Australia, although all eight States and Territories offer maintenance OST in prison, only five of these (NSW included) allow first-time initiation of OST in prison [54]. Despite NSW having one of the largest in-prison OST programs in Australia [55], culturally relevant and accessible programs for Indigenous people in the community are still needed.

Indigenous males were poorly retained in their first OST treatment episode. Given that Indigenous males more frequently commenced OST treatment in prison, poor treatment continuity at the time of release is likely to have resulted in shorter treatment episodes. This highlights the need for the development and implementation of evidence-based and culturally tailored interventions to support the transition of Indigenous males from prison to the community. This is important because maintaining retention in OST is necessary to achieve optimal OST treatment outcomes [19]. About a quarter of Indigenous people aged 15 years and over living in non-remote areas report difficulty accessing health services, a rate ten times that of the general Australian population [56]. Although efforts have been made to close the gap in health and disease burden between Indigenous and non-Indigenous Australians, Indigenous men still have the poorest health outcomes [47]. It is known that Indigenous people are more likely to access services in prison [57], and prisons provide an important opportunity for the provision of health care and screening to Indigenous people that would otherwise have difficulty accessing health services [58]. There remains a need to further examine issues around gender and access to OST among Indigenous people in order to maximise treatment outcomes.

### Strengths and limitations

The cohort in this study was defined on the basis that individuals had a recorded history of opioid dependence, recorded Indigenous status (Indigenous/non-Indigenous), and at least one criminal charge in NSW during the study period. Through the use of state-wide administrative datasets, this study presented a unique opportunity to evaluate longitudinal population-level data. Given that NSW has the largest proportion of Indigenous residents in Australia, and also the largest proportion of clients in OST, a major strength of our study is that the cohort is representative of a large number of Indigenous and non-Indigenous opioid-dependent people in the Australian population. However, as the provision of OST and criminal justice services vary between States and Territories, the findings of this study may not be the same across jurisdictions. In addition, although the National Deaths Index is able to capture deaths occurring in any State or Territory in Australia, the other datasets used in this study are NSW specific. Therefore, we are not able to capture events that occurred outside NSW or follow those individuals who might have migrated to other States or outside Australia. Furthermore, although Australian OST services aim to ensure access to disadvantaged populations including Indigenous populations [59], rates of participation in the community are not consistent across Australia [60]. Hence, the results may not necessarily be generalisable to other Indigenous opioid-dependent populations.

Information regarding the identity of Indigenous Australians is not always reliable in health datasets; missing data is common, and Aboriginal and Torres Strait Islander people are often under-counted [61]. Furthermore, estimates of disparities in health between Indigenous and non-Indigenous people can be affected by the way in which Indigenous identity is recorded [62]. The ever-identified strategy used in this study meant that Aboriginal and Torres Strait Islander people were included despite changes in administrative data recording or personal preference to identify as Indigenous or not over time [63]. Although recent studies have suggested that the application of other approaches to identify Aboriginal or Torres Strait Islander people in datasets may have improved accuracy [62], we were restricted to the approach used by the data custodians in constructing the datasets. However, we endeavoured to improve the accuracy of correctly identifying Indigenous people in the cohort by triangulating information from two data sources.

Rates of offending based on data from administrative datasets provide estimates of the number of charges which are made, based on offences which are reported to correctional authorities. Consequently, offences which are unreported are not able to be enumerated, which may lead to an under-estimation of actual rates of offending. Hence, the rates of offending reported in our study are also likely to have been under-estimated. As the main purpose of this study was to undertake a detailed comparison of offending, time in custody, and OST treatment utilisation among Indigenous and non-Indigenous people in an opioid-dependent population, we did not examine whether time in OST directly affects either offending or time in custody, but is an important area for future research. Also, given that we were unable to assess the temporal relationship between time of offence and initiation of opioid use, this presents another area for future research.

## Conclusions

There are clear differences in the nature and levels of offending, as well as time spent in custody among Indigenous and non-Indigenous people with a history of opioid dependence and contact with the criminal justice system. Indigenous populations continue to experience social exclusion and ongoing inequity across determinants of health and our findings highlight that Indigenous people with opioid use disorders are further disadvantaged. Although the prison setting appears to be an important access point for OST among Indigenous people, the under-treatment of Indigenous people in the community is also apparent, and there appears to be a lack of continuity between the prison and community OST systems. The underlying reasons for these differences therefore need to be addressed, with a focus on the development, implementation and rigorous evaluation of more targeted and evidence-based culturally tailored interventions.

## Electronic supplementary material

Additional file 1: **Outcomes of charges for opioid dependent people with at least one criminal charge by Indigenous status and sex, December 1993- December 2011.** (DOC 66 KB)

Additional file 2: **Number of charges against opioid dependent people per 100 person years for non-Indigenous and Indigenous males, according to category of offence, December 1993-December 2011.** (DOC 276 KB)

Additional file 3: **Number of charges against opioid dependent people per 100 person years for non-Indigenous and Indigenous females, according to category of offence, December 1993 – December 2011.** (DOC 270 KB)

Additional file 4: **Comparison of age of first offence and age of first treatment entry – n=2,815.** (DOC 38 KB)
