# Supplementary material for: Offending, custody and opioid substitution therapy treatment utilisation among opioid-dependent people in contact with the criminal justice system: comparison of Indigenous and non-Indigenous Australians
Source: BMC Public Health. 2014 Sep 6;14:920. doi: 10.1186/1471-2458-14-920 (PMC4168057; doi:10.1186/1471-2458-14-920)
Supplement: Supplementary file 1 — Additional file 1: Outcomes of charges for opioid dependent people with at least one criminal charge by Indigenous status and sex, December 1993- December 2011. (DOC 66 KB) [file 12889_2014_7046_MOESM1_ESM.doc]

**Additional file 1. Outcomes of charges for opioid dependent people with at least one criminal charge by Indigenous status and sex, December 1993- December 2011**

|  |  | **Indigenous**  **(N = 6,830)** | | | **Non-Indigenous**  **(N = 28,132)** | | | |
| --- | --- | --- | --- | --- | --- | --- | --- | --- |
|  | **Males**  **(N = 4,615)** | | **Females**  **(N = 2,215)** | | **Males**  **(N = 20,179)** | | **Females**  **(N = 7,953)** | |
| **Offence type** | **N**  **charges** | **%**  **proven** | **N**  **charges** | **%**  **proven** | **N**  **charges** | **%**  **proven** | **N**  **charges** | **%**  **proven** |
| Homicide and related offences | 113 | 52.2 | 31 | 54.8 | 229 | 51.1 | 33 | 51.5 |
| Acts intended to cause injury | 21,806 | 69.6 | 5,841 | 77.0 | 30,069 | 68.5 | 5,421 | 73.9 |
| Sexual assault and related offences | 571 | 55.2 | 19 | 57.9 | 717 | 52.0 | 22 | 31.8 |
| Dangerous or negligent acts endangering persons | 3,270 | 83.5 | 564 | 86.7 | 8,820 | 84.6 | 1,678 | 86.4 |
| Abduction, other offences against the person | 1,048 | 76.9 | 156 | 71.8 | 1,468 | 73.2 | 129 | 67.4 |
| Robbery, extortion and related offences | 2,444 | 73.2 | 566 | 71.2 | 3,184 | 76.4 | 370 | 72.4 |
| Unlawful entry/burglary, break and enter | 11,483 | 80.0 | 1,686 | 76.1 | 18,419 | 81.0 | 1,928 | 77.4 |
| Theft and related offences | 33,619 | 84.0 | 16,745 | 88.1 | 80,705 | 85.4 | 24,748 | 88.8 |
| Fraud, deception and related offences | 4,189 | 88.6 | 3,086 | 89.7 | 15,316 | 88.9 | 7,760 | 90.8 |
| Illicit drug offences | 10,999 | 90.6 | 4,133 | 89.6 | 38,303 | 89.9 | 9,600 | 88.8 |
| Prohibited and regulated weapons offences | 1,541 | 80.6 | 239 | 78.2 | 4,662 | 78.4 | 449 | 77.1 |
| Property damage and environmental pollution | 7,977 | 84.8 | 1,699 | 87.7 | 11,227 | 84.0 | 1,737 | 84.2 |
| Public order offences | 16,227 | 83.5 | 5,402 | 89.8 | 28,657 | 85.4 | 6,342 | 90.8 |
| Traffic and vehicle regulatory offences | 21,107 | 94.7 | 6,124 | 94.8 | 60,887 | 94.6 | 14,795 | 95.0 |
| Offences against justice procedures | 19,485 | 85.3 | 6,591 | 88.1 | 32,875 | 86.4 | 8,065 | 86.9 |
| Miscellaneous offences | 1,179 | 85.7 | 653 | 84.4 | 4,200 | 81.5 | 1,388 | 82.1 |
| **Any property offence** | 36,966 | 84.9 | 15,628 | 89.4 | 79,895 | 87.7 | 25,035 | 90.9 |
| **Any violent offence** | 24,898 | 69.6 | 6,451 | 76.3 | 34,122 | 68.7 | 5,840 | 73.5 |
| **Total** | **157,058** | **83.5%** | **53,535** | **87.3%** | **339,738** | **85.6%** | **84,465** | **88.4%** |
