# Supplementary material for: Offending, custody and opioid substitution therapy treatment utilisation among opioid-dependent people in contact with the criminal justice system: comparison of Indigenous and non-Indigenous Australians
Source: BMC Public Health. 2014 Sep 6;14:920. doi: 10.1186/1471-2458-14-920 (PMC4168057; doi:10.1186/1471-2458-14-920)
Supplement: Supplementary file 3 — Additional file 3: Number of charges against opioid dependent people per 100 person years for non-Indigenous and Indigenous females, according to category of offence, December 1993 – December 2011. (DOC 270 KB) [file 12889_2014_7046_MOESM3_ESM.doc]

**Additional file 3. Number of charges against opioid dependent people per 100 person years for non-Indigenous and Indigenous females, according to category of offence, December 1993 – December 2011**

**
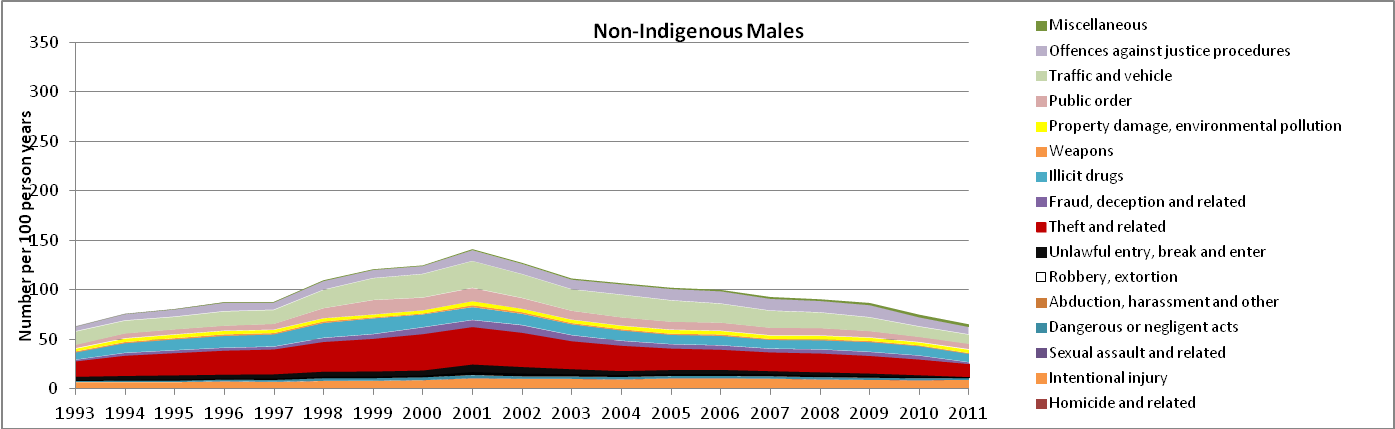

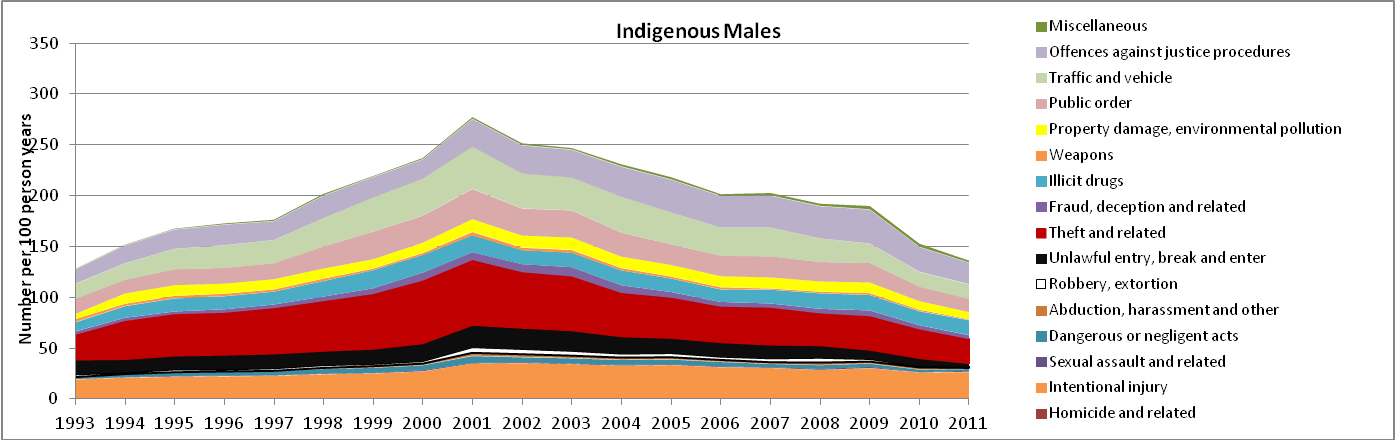
**
