# Supplementary material for: Offending, custody and opioid substitution therapy treatment utilisation among opioid-dependent people in contact with the criminal justice system: comparison of Indigenous and non-Indigenous Australians
Source: BMC Public Health. 2014 Sep 6;14:920. doi: 10.1186/1471-2458-14-920 (PMC4168057; doi:10.1186/1471-2458-14-920)
Supplement: Supplementary file 4 — Additional file 4: Comparison of age of first offence and age of first treatment entry – n=2,815. (DOC 38 KB) [file 12889_2014_7046_MOESM4_ESM.doc]

**Additional file 4. Comparison of age of first offence and age of first treatment entry – n=2,815**

|  | **Males (N=1,877)** | | | |  | **Females (N=938)** | | | |  |
| --- | --- | --- | --- | --- | --- | --- | --- | --- | --- | --- |
|  | **Indigenous**  **(N=745)** | | **Non-Indigenous**  **(N=1132)** | |  | **Indigenous**  **(N=342)** | | **Non-Indigenous**  **(N=596)** | |  |
|  | **Median** | **IQR** | **Median** | **IQR** | ***P*** | **Median** | **IQR** | **Median** | **IQR** | ***P*** |
| **Age of first offence (years)** | 14.6 | 3.6 | 17.5 | 14.4 | <0.001 | 15.8 | 4.0 | 18.8 | 4.6 | <0.001 |
| **Age of first treatment entry (years)** | 21.2 | 3.6 | 21.4 | 2.6 | 0.1580 | 20.0 | 3.7 | 20.3 | 4.2 | 0.0114 |
| **Difference between treatment entry and first age of offence (years)** | 6.6 | N/A | 3.9 | N/A | N/A | 4.2 | N/A | 1.5 | N/A | N/A |
